# 3-Chloro-6-[4-(2-pyrid­yl)piperazin-1-yl]pyridazine

**DOI:** 10.1107/S1600536809050727

**Published:** 2009-12-04

**Authors:** Hakan Arslan, Semra Utku, Kenneth I. Hardcastle, Mehtap Gökçe, Sheri Lense

**Affiliations:** a Department of Chemistry, Emory University, Atlanta, GA-30322, USA; b Department of Chemistry, Faculty of Pharmacy, Mersin University, Mersin TR-33169, Turkey; c Department of Pharmaceutical Chemistry, Faculty of Pharmacy, Mersin University, Mersin TR-33169, Turkey; d Department of Pharmaceutical Chemistry, Faculty of Pharmacy, Gazi University, Ankara TR-06330, Turkey

## Abstract

In the title compound, C_13_H_14_ClN_5_, the piperazine ring adopts a chair conformation  and the dihedral angle between the aromatic rings is 13.91 (7)°. The crystal structure is stabilized by weak inter­molecular C—H⋯N hydrogen-bond inter­actions.

## Related literature

For the synthesis, structures and analgesic and anti-inflammatory activity of substituted pyridazine derivatives, see: Boissier *et al.* (1963 ▶); Gokce *et al.* (2001 ▶, 2004 ▶, 2005 ▶, 2009 ▶); Sahin *et al.* (2004 ▶); Dundar *et al.* (2007 ▶). For general background to non-opioid analgesic derivatives, see: Sato *et al.* (1981 ▶); Banoglu *et al.* (2004 ▶); Giovannoni *et al.* (2003 ▶)·For puckering parameters, see: Cremer & Pople (1975 ▶).
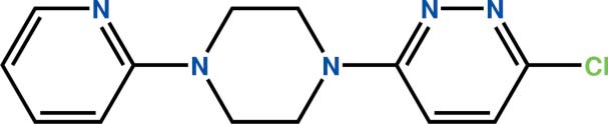


## Experimental

### 

#### Crystal data

C_13_H_14_ClN_5_
*M* *_r_* = 275.74
Triclinic, 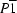

*a* = 5.912 (3) Å
*b* = 8.088 (5) Å
*c* = 13.689 (8) Å
α = 83.359 (9)°
β = 83.019 (9)°
γ = 75.168 (9)°
*V* = 625.5 (6) Å^3^
*Z* = 2
Mo *K*α radiation
μ = 0.30 mm^−1^
*T* = 296 K
0.16 × 0.15 × 0.14 mm

#### Data collection

Bruker APEXII CCD diffractometer
Absorption correction: multi-scan (*SADABS*; Bruker, 2008 ▶) *T*_min_ = 0.954, *T*_max_ = 0.959
11395 measured reflections
3275 independent reflections
2264 reflections with *I* > 2σ(*I*)
*R*_int_ = 0.049

#### Refinement

*R*[*F* ^2^ > 2σ(*F* ^2^)] = 0.039
*wR*(*F* ^2^) = 0.095
*S* = 0.95
3275 reflections
172 parameters
H-atom parameters constrained
Δρ_max_ = 0.27 e Å^−3^
Δρ_min_ = −0.23 e Å^−3^

### 

Data collection: *APEX2* (Bruker (2008 ▶); cell refinement: *SAINT* (Bruker, 2008 ▶); data reduction: *SAINT*; program(s) used to solve structure: *SHELXS97* (Sheldrick, 2008 ▶); program(s) used to refine structure: *SHELXL97* (Sheldrick, 2008 ▶); molecular graphics: *SHELXTL* (Sheldrick, 2008 ▶); software used to prepare material for publication: *SHELXTL*.

## Supplementary Material

Crystal structure: contains datablocks I, global. DOI: 10.1107/S1600536809050727/hg2610sup1.cif

Structure factors: contains datablocks I. DOI: 10.1107/S1600536809050727/hg2610Isup2.hkl

Additional supplementary materials:  crystallographic information; 3D view; checkCIF report

## Figures and Tables

**Table 1:** Hydrogen-bond geometry (Å, °)

*D*—H⋯*A*	*D*—H	H⋯*A*	*D*⋯*A*	*D*—H⋯*A*
C3—H3⋯N1^i^	0.93	2.58	3.346 (3)	140

Symmetry code: (i) 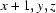.

**Fig. 1.**
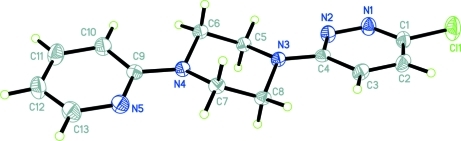
The molecular structure of (I), showing ellipsoids at the 50% probability level.

**Fig. 2.**
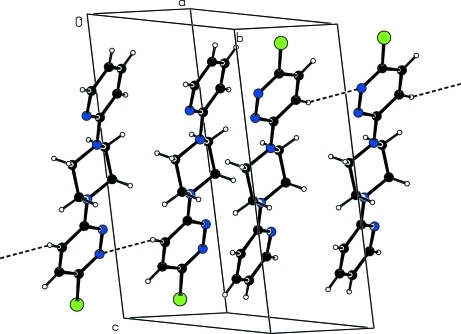
The molecular packing of (I). The hydrogen bonds are shown as dashed lines.

**Fig. 3.**
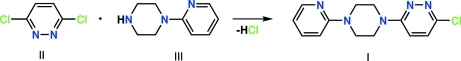
Preparation of compound (I)

## supplementary crystallographic information

### Comment

The opioid derivative analgesics have significant side effects, therefore, the current research is focused on non-opioid analgesics that do not have serious side effects but are as effective as the opioids. One of the non-opioid analgesic derivatives is emorfazone which has a substituted pyridazine (Sato *et al.*, 1981). In addition, some pyridazinone derivatives bearing an alkylpiperazinyl alkyl moiety also show interesting antinociceptive activity (Banoglu *et al.*, 2004; Giovannoni *et al.*, 2003).

Recently, our team focused on the synthesis, characterization, and analgesics-anti-inflammatory activity of substituted pyridazine derivatives (Dundar *et al.*, 2007; Gokce *et al.*, 2001, 2004, 2005, 2009; Sahin *et al.*, 2004). The compound, 3-chloro-6-(4-pyridin-2-ylpiperazin-1-yl)pyridazine, (I), Scheme 1, is one example and in this article we report on the crystal structure of the title compound, Figure 1.

The molecular structure of (I) consists of 3-chloropyridazine and pyridine arms connected to a piperazine ring. The 3-chloropyridazine and pyridine rings are planar with a maximum deviation of 0.006 (1) Å for atom C4 and -0.014 (2) Å for atom C12. The dihedral angle between these two rings is 13.91 (7) °. The piperazine ring adopts a chair conformation. This is confirmed by the puckering parameters *q*_2_ = 0.0056 (13) Å, *q*_3_ = -0.5388 (13) Å, *Q*_T_ = 0.5388 (13) Å, *θ* = 179.67 (14) ° and φ = 221 (14) ° (Cremer & Pople, 1975).

The conformations of the 3-chloropyridazine and pyridine rings are best described by the torsion angles of -155.41 (12) ° and 156.51 (12) ° for C4—N3—C5—C6 and C9—N4—C7—C8, respectively; thus they adopt - antiperiplanar and + antiperiplanar conformations, respectively.

The crystal packing is dominated by weak intermolecular C3—H3···N1 (1 + *x*, *y*, *z*) hydrogen bonds, with H···N = 2.58 Å and a C—H···N angle of 140 ° (Figure 2).

### Experimental

A mixture of 3,6-dichloropyridazine, (II), (1.7 mol) and 1-(2-pyridyl)piperazine, (III), (2.0 mol) in ethanol (10 ml) was heated under reflux for 4 h after which the mixture was cooled to room temperature (Figure 3) (Boissier *et al.*, 1963). The resulting crude precipitate was filtered off and purified by repeated washing with small portions of cold ethanol. The precipitate formed was crystallized from CH_2_Cl_2_:ethanol (5:10) to give the compound 3-chloro-6-(4-pyridin-2-ylpiperazin-1-yl) pyridazine, (I), as white crystals. Yields: 0.270 g, 58%. *M*.p.: 153 °C. ^1^H NMR (DMSO-d_6_) δ: 8.16–8.13 (d, 1H, pyridyl), 7.63–7.56 (m, 2H, pyridyl), 7.47–7.44 (d, 1H, pyridazin), 6.93–6.89 (d, 1H, pyridyl), 6.75–6.66 (d, 1H, pyridazin), 3.73–3.62(m, 4H, piperazine), 3.17–3.14 (m, 4H, piperazine). MS (EI) m/*z*: 276 (*M*^+^). Anal. Calc. for C_13_H_14_N_5_Cl: C, 56.63; H, 5.12; N, 25.40%. Found: C, 56.60; H, 5.10; N, 24.42%.

### Refinement

The H atoms were positioned geometrically and allowed to ride on their parent atoms, with C—H distances of 0.93 Å (CH) or 0.97 Å (CH_2_), and with *U*_iso_(H) = 1.2*U*_eq_ of the parent atoms.

### Crystal data

**Table:**

C_13_H_14_ClN_5_	*Z* = 2
*M**_r_* = 275.74	*F*(000) = 288
Triclinic, *P*1	*D*_x_ = 1.464 Mg m^−^^3^
Hall symbol: -P 1	Mo *K*α radiation, λ = 0.71073 Å
*a* = 5.912 (3) Å	Cell parameters from 2718 reflections
*b* = 8.088 (5) Å	θ = 2.6–28.2°
*c* = 13.689 (8) Å	µ = 0.30 mm^−^^1^
α = 83.359 (9)°	*T* = 296 K
β = 83.019 (9)°	Block, colourless
γ = 75.168 (9)°	0.16 × 0.15 × 0.14 mm
*V* = 625.5 (6) Å^3^	

### Data collection

**Table:**

Bruker APEXII CCD diffractometer	3275 independent reflections
Radiation source: fine-focus sealed tube	2264 reflections with *I* > 2σ(*I*)
graphite	*R*_int_ = 0.049
φ and ω scans	θ_max_ = 29.0°, θ_min_ = 1.5°
Absorption correction: multi-scan (*SADABS*; Bruker, 2008)	*h* = −8→7
*T*_min_ = 0.954, *T*_max_ = 0.959	*k* = −10→11
11395 measured reflections	*l* = −18→18

### Refinement

**Table:**

Refinement on *F*^2^	Primary atom site location: structure-invariant direct methods
Least-squares matrix: full	Secondary atom site location: difference Fourier map
*R*[*F*^2^ > 2σ(*F*^2^)] = 0.039	Hydrogen site location: inferred from neighbouring sites
*wR*(*F*^2^) = 0.095	H-atom parameters constrained
*S* = 0.95	*w* = 1/[σ^2^(*F*_o_^2^) + (0.0433*P*)^2^] where *P* = (*F*_o_^2^ + 2*F*_c_^2^)/3
3275 reflections	(Δ/σ)_max_ = 0.001
172 parameters	Δρ_max_ = 0.27 e Å^−^^3^
0 restraints	Δρ_min_ = −0.23 e Å^−^^3^

### Special details

**Table:**

Geometry. All e.s.d.'s (except the e.s.d. in the dihedral angle between two l.s. planes) are estimated using the full covariance matrix. The cell e.s.d.'s are taken into account individually in the estimation of e.s.d.'s in distances, angles and torsion angles; correlations between e.s.d.'s in cell parameters are only used when they are defined by crystal symmetry. An approximate (isotropic) treatment of cell e.s.d.'s is used for estimating e.s.d.'s involving l.s. planes.
Refinement. Refinement of *F*^2^ against ALL reflections. The weighted *R*-factor *wR* and goodness of fit *S* are based on *F*^2^, conventional *R*-factors *R* are based on *F*, with *F* set to zero for negative *F*^2^. The threshold expression of *F*^2^ > σ(*F*^2^) is used only for calculating *R*-factors(gt) *etc*. and is not relevant to the choice of reflections for refinement. *R*-factors based on *F*^2^ are statistically about twice as large as those based on *F*, and *R*- factors based on ALL data will be even larger.

### Fractional atomic coordinates and isotropic or equivalent isotropic displacement parameters (Å^2^)

**Table:**

	*x*	*y*	*z*	*U*_iso_*/*U*_eq_	
C1	0.2600 (3)	1.08679 (18)	0.14549 (10)	0.0258 (3)	
C2	0.4977 (3)	1.05027 (18)	0.15814 (10)	0.0262 (3)	
H2	0.6045	1.0856	0.1098	0.031*	
C3	0.5676 (2)	0.96086 (18)	0.24396 (10)	0.0233 (3)	
H3	0.7248	0.9314	0.2563	0.028*	
C4	0.3929 (2)	0.91394 (16)	0.31412 (10)	0.0183 (3)	
C5	0.2554 (2)	0.78257 (18)	0.47211 (9)	0.0191 (3)	
H5A	0.2447	0.6710	0.4565	0.023*	
H5B	0.1066	0.8639	0.4613	0.023*	
C6	0.2997 (2)	0.77262 (18)	0.57925 (10)	0.0201 (3)	
H6A	0.2902	0.8869	0.5973	0.024*	
H6B	0.1795	0.7281	0.6206	0.024*	
C7	0.7198 (2)	0.71363 (18)	0.53092 (9)	0.0195 (3)	
H7A	0.8676	0.6312	0.5417	0.023*	
H7B	0.7325	0.8246	0.5467	0.023*	
C8	0.6760 (2)	0.72468 (17)	0.42325 (10)	0.0191 (3)	
H8A	0.7957	0.7701	0.3822	0.023*	
H8B	0.6860	0.6107	0.4047	0.023*	
C9	0.5772 (2)	0.58513 (17)	0.69070 (10)	0.0195 (3)	
C10	0.3966 (3)	0.57581 (19)	0.76644 (11)	0.0273 (3)	
H10	0.2411	0.6307	0.7571	0.033*	
C11	0.4545 (3)	0.4840 (2)	0.85437 (11)	0.0326 (4)	
H11	0.3373	0.4754	0.9052	0.039*	
C12	0.6857 (3)	0.4044 (2)	0.86779 (11)	0.0335 (4)	
H12	0.7277	0.3393	0.9263	0.040*	
C13	0.8513 (3)	0.4253 (2)	0.79115 (11)	0.0317 (4)	
H13	1.0080	0.3743	0.8002	0.038*	
Cl1	0.15611 (8)	1.19962 (6)	0.03723 (3)	0.04376 (16)	
N1	0.1002 (2)	1.04206 (16)	0.21036 (9)	0.0273 (3)	
N2	0.1664 (2)	0.95301 (15)	0.29644 (8)	0.0240 (3)	
N3	0.44321 (19)	0.83591 (14)	0.40660 (8)	0.0185 (3)	
N4	0.53064 (19)	0.66186 (14)	0.59658 (8)	0.0190 (3)	
N5	0.8031 (2)	0.51407 (15)	0.70410 (9)	0.0259 (3)	

### Atomic displacement parameters (Å^2^)

**Table:**

	*U*^11^	*U*^22^	*U*^33^	*U*^12^	*U*^13^	*U*^23^
C1	0.0272 (8)	0.0267 (8)	0.0218 (7)	−0.0052 (6)	−0.0029 (6)	0.0016 (6)
C2	0.0253 (8)	0.0305 (8)	0.0236 (7)	−0.0119 (7)	0.0053 (6)	−0.0019 (6)
C3	0.0171 (7)	0.0278 (8)	0.0256 (7)	−0.0084 (6)	0.0024 (6)	−0.0035 (6)
C4	0.0171 (7)	0.0169 (7)	0.0215 (7)	−0.0052 (5)	0.0011 (5)	−0.0050 (5)
C5	0.0120 (7)	0.0230 (7)	0.0219 (7)	−0.0054 (6)	0.0007 (5)	−0.0006 (5)
C6	0.0129 (7)	0.0240 (7)	0.0220 (7)	−0.0033 (6)	0.0015 (5)	−0.0015 (6)
C7	0.0121 (7)	0.0226 (7)	0.0239 (7)	−0.0057 (6)	−0.0001 (5)	−0.0010 (5)
C8	0.0107 (7)	0.0217 (7)	0.0239 (7)	−0.0031 (5)	0.0017 (5)	−0.0028 (5)
C9	0.0198 (7)	0.0182 (7)	0.0219 (7)	−0.0057 (6)	−0.0031 (6)	−0.0033 (5)
C10	0.0208 (8)	0.0327 (8)	0.0262 (8)	−0.0048 (7)	−0.0005 (6)	0.0011 (6)
C11	0.0320 (9)	0.0415 (9)	0.0236 (8)	−0.0123 (8)	0.0016 (7)	0.0023 (7)
C12	0.0369 (10)	0.0389 (9)	0.0240 (8)	−0.0077 (8)	−0.0103 (7)	0.0045 (7)
C13	0.0246 (9)	0.0378 (9)	0.0314 (8)	−0.0030 (7)	−0.0101 (7)	0.0000 (7)
Cl1	0.0423 (3)	0.0562 (3)	0.0281 (2)	−0.0095 (2)	−0.00750 (18)	0.01386 (18)
N1	0.0230 (7)	0.0329 (7)	0.0246 (6)	−0.0060 (6)	−0.0038 (5)	0.0034 (5)
N2	0.0166 (6)	0.0297 (7)	0.0242 (6)	−0.0054 (5)	−0.0023 (5)	0.0033 (5)
N3	0.0115 (6)	0.0229 (6)	0.0198 (6)	−0.0039 (5)	0.0009 (4)	0.0001 (5)
N4	0.0119 (6)	0.0230 (6)	0.0209 (6)	−0.0042 (5)	0.0003 (5)	0.0007 (5)
N5	0.0201 (7)	0.0307 (7)	0.0259 (7)	−0.0037 (5)	−0.0052 (5)	−0.0014 (5)

### Geometric parameters (Å, °)

**Table:**

C1—N1	1.3071 (19)	C7—H7A	0.9700
C1—C2	1.388 (2)	C7—H7B	0.9700
C1—Cl1	1.7401 (16)	C8—N3	1.4661 (18)
C2—C3	1.3574 (19)	C8—H8A	0.9700
C2—H2	0.9300	C8—H8B	0.9700
C3—C4	1.4170 (18)	C9—N5	1.3377 (18)
C3—H3	0.9300	C9—N4	1.3909 (17)
C4—N2	1.3401 (18)	C9—C10	1.405 (2)
C4—N3	1.3784 (17)	C10—C11	1.372 (2)
C5—N3	1.4617 (17)	C10—H10	0.9300
C5—C6	1.5104 (19)	C11—C12	1.378 (2)
C5—H5A	0.9700	C11—H11	0.9300
C5—H5B	0.9700	C12—C13	1.372 (2)
C6—N4	1.4582 (18)	C12—H12	0.9300
C6—H6A	0.9700	C13—N5	1.3402 (18)
C6—H6B	0.9700	C13—H13	0.9300
C7—N4	1.4635 (17)	N1—N2	1.3534 (16)
C7—C8	1.5159 (19)		
			
N1—C1—C2	124.64 (13)	N3—C8—C7	110.54 (11)
N1—C1—Cl1	115.24 (12)	N3—C8—H8A	109.5
C2—C1—Cl1	120.12 (11)	C7—C8—H8A	109.5
C3—C2—C1	117.20 (13)	N3—C8—H8B	109.5
C3—C2—H2	121.4	C7—C8—H8B	109.5
C1—C2—H2	121.4	H8A—C8—H8B	108.1
C2—C3—C4	117.76 (14)	N5—C9—N4	116.30 (12)
C2—C3—H3	121.1	N5—C9—C10	121.67 (13)
C4—C3—H3	121.1	N4—C9—C10	121.97 (13)
N2—C4—N3	116.16 (11)	C11—C10—C9	118.58 (14)
N2—C4—C3	121.66 (12)	C11—C10—H10	120.7
N3—C4—C3	122.04 (13)	C9—C10—H10	120.7
N3—C5—C6	111.31 (11)	C10—C11—C12	120.29 (14)
N3—C5—H5A	109.4	C10—C11—H11	119.9
C6—C5—H5A	109.4	C12—C11—H11	119.9
N3—C5—H5B	109.4	C13—C12—C11	117.17 (14)
C6—C5—H5B	109.4	C13—C12—H12	121.4
H5A—C5—H5B	108.0	C11—C12—H12	121.4
N4—C6—C5	110.91 (11)	N5—C13—C12	124.63 (14)
N4—C6—H6A	109.5	N5—C13—H13	117.7
C5—C6—H6A	109.5	C12—C13—H13	117.7
N4—C6—H6B	109.5	C1—N1—N2	119.04 (12)
C5—C6—H6B	109.5	C4—N2—N1	119.68 (11)
H6A—C6—H6B	108.0	C4—N3—C5	118.35 (11)
N4—C7—C8	111.62 (11)	C4—N3—C8	121.18 (11)
N4—C7—H7A	109.3	C5—N3—C8	112.41 (11)
C8—C7—H7A	109.3	C9—N4—C6	120.47 (11)
N4—C7—H7B	109.3	C9—N4—C7	118.98 (11)
C8—C7—H7B	109.3	C6—N4—C7	112.49 (11)
H7A—C7—H7B	108.0	C9—N5—C13	117.56 (12)
			
N1—C1—C2—C3	0.4 (2)	C3—C4—N3—C5	−176.37 (12)
Cl1—C1—C2—C3	−179.57 (11)	N2—C4—N3—C8	154.15 (12)
C1—C2—C3—C4	−0.8 (2)	C3—C4—N3—C8	−30.0 (2)
C2—C3—C4—N2	1.2 (2)	C6—C5—N3—C4	−155.41 (12)
C2—C3—C4—N3	−174.44 (13)	C6—C5—N3—C8	55.41 (15)
N3—C5—C6—N4	−54.49 (15)	C7—C8—N3—C4	157.23 (12)
N4—C7—C8—N3	53.56 (15)	C7—C8—N3—C5	−54.57 (15)
N5—C9—C10—C11	−3.3 (2)	N5—C9—N4—C6	−167.32 (12)
N4—C9—C10—C11	173.85 (13)	C10—C9—N4—C6	15.4 (2)
C9—C10—C11—C12	0.6 (2)	N5—C9—N4—C7	−20.90 (18)
C10—C11—C12—C13	1.6 (3)	C10—C9—N4—C7	161.84 (13)
C11—C12—C13—N5	−1.5 (3)	C5—C6—N4—C9	−156.99 (12)
C2—C1—N1—N2	−0.3 (2)	C5—C6—N4—C7	54.58 (15)
Cl1—C1—N1—N2	179.64 (10)	C8—C7—N4—C9	156.51 (12)
N3—C4—N2—N1	174.73 (12)	C8—C7—N4—C6	−54.56 (15)
C3—C4—N2—N1	−1.2 (2)	N4—C9—N5—C13	−173.88 (13)
C1—N1—N2—C4	0.7 (2)	C10—C9—N5—C13	3.4 (2)
N2—C4—N3—C5	7.76 (18)	C12—C13—N5—C9	−1.0 (2)

### Hydrogen-bond geometry (Å, °)

**Table:**

*D*—H···*A*	*D*—H	H···*A*	*D*···*A*	*D*—H···*A*
C3—H3···N1^i^	0.93	2.58	3.346 (3)	140

Symmetry codes: (i) *x*+1, *y*, *z*.
